# Silver Sucrose Octasulfate (IASOS™) as a Valid Active Ingredient into a Novel Vaginal Gel against Human Vaginal Pathogens: *In Vitro* Antimicrobial Activity Assessment

**DOI:** 10.1371/journal.pone.0097791

**Published:** 2014-06-04

**Authors:** Cinzia Marianelli, Paola Petrucci, Maria Cristina Comelli, Gabriella Calderini

**Affiliations:** 1 Department of Food Safety and Veterinary Public Health, Istituto Superiore di Sanità, Rome, Italy; 2 CM&D Pharma Srl, a wholly owned subsidiary of CM&D Pharma Limited, Padova, Italy; University of Illinois, United States of America

## Abstract

This *in vitro* study assessed the antimicrobial properties of a novel octasilver salt of Sucrose Octasulfate (IASOS) as well as of an innovative vaginal gel containing IASOS (SilSOS Femme), against bacterial and yeast pathogens isolated from human clinical cases of symptomatic vaginal infections. In BHI and LAPT culture media, different ionic silver concentrations and different pHs were tested. IASOS exerted a strong antimicrobial activity towards all the pathogens tested in both culture media. The results demonstrated that salts and organic compounds present in the culture media influenced IASOS efficacy only to a moderate extent. Whereas comparable MBCs (Minimal Bactericidal Concentrations) were observed for *G. vaginalis* (10 mg/L Ag^+^), *E. coli* and *E. aerogenes* (25 mg/L Ag^+^) in both media, higher MBCs were found for *S*. *aureus* and *S. agalactiae* in LAPT cultures (50 mg/L Ag^+^ versus 25 mg/L Ag^+^). No minimal concentration totally inhibiting the growth of *C. albicans* was found. Nevertheless, in both media at the highest ionic silver concentrations (50–200 mg/L Ag^+^), a significant 34–52% drop in *Candida* growth was observed. pH differently affected the antimicrobial properties of IASOS against bacteria or yeasts; however, a stronger antimicrobial activity at pH higher than the physiological pH was generally observed. It can be therefore concluded that IASOS exerts a bactericidal action against all the tested bacteria and a clear fungistatic action against *C. albicans*. The antimicrobial activity of the whole vaginal gel SilSOS Femme further confirmed the antimicrobial activity of IASOS. Overall, our findings support IASOS as a valid active ingredient into a vaginal gel.

## Introduction

Vaginal infections are the most common cause of women discomfort and complain, occurring in about 10% of pre-puberty girls, 90% of women of reproductive age and in 40–50% of post-menopausal women [1]. Vaginal infections may be both symptomatic and asymptomatic. Symptomatic infections can present with erythema, oedema, itchiness, burning, pain and vaginal discharge. Vulva may also be involved.

Pathogens responsible for vaginal infections include bacteria, fungi and protozoa. Bacterial vaginosis (BV), aerobic vaginitis (AV) and candidiasis are the most common infections in women of childbearing age, characterised by an imbalanced vaginal microbiota population and vaginal discharge. Whereas BV, often asymptomatic and characterised by a discharge of typical fishy odour, is a polymicrobial syndrome with a variety of opportunistic pathogenic bacteria such as *Gardnerella vaginalis*, replacing the resident *Lactobacillus* species [2], [3], [4], in AV the resident lactobacilli are replaced by intestinally-derived aerobic microorganisms, such as *Escherichia coli*, *Streptococcus agalactiae, Staphylococcus aureus* and *Enterococcus faecalis* [5], [6]. An highly symptomatic inflammatory state, AV typically presents with a yellow, smelly vaginal discharge. BV and AV are generally accompanied with a vaginal pH increase above 4.5, beyond the healthy vaginal pH of 3.8–4.5 [5], [7].

Candidiasis is the most frequent symptomatic mycotic infection caused by the yeast *Candida albicans* and a few other pathogenic *Candida* species. During their lifetime, 75% of all women are expected to experience at least one episode of Candida-induced vaginitis [8], [9]. Acute pruritus and smelly vaginal discharge are usual complains in vaginal candidiasis, associated with vaginal discomfort, irritation, vulvar burning, dyspareunia and dysuria. Differently from BV and AV, the vaginal pH usually remains in the normal range [9].

The common therapeutic strategy for vaginal infections is an antibiotic treatment [6], [10], [11]. Variable sensitivity or resistance to different antimicrobial agents have been reported in time, thus challenging treatment efficacy and frequently resulting in relapses [12], [13], [14]. Vaginal pathogens may also survive antibiotics through the formation of bacterial colonies on the vaginal epithelium surface and named biofilms, also reported to be responsible for vaginal infection recurrences [15], [16].

The magnitude of gynaecological and obstetrical consequences of vaginal infections, particularly severe in pregnant women [17], [18] recently fostered the development of newly emerging therapies, including either novel vaginal delivery systems aimed to prolong the residence time of drugs into the vaginal cavity, or novel antimicrobial agents. Nifuratel [19], [20], plant-derived essential oils [21], [22], glycerol monolaurate [23] and silver [11] are just a few examples of emerging strategies with a good safety profile.

Silver is well known for its strong antimicrobial properties. Silver-based biocides, including both ionic silver forms and metallic silver nanoparticles are antimicrobial towards a broad spectrum of Gram-negative and Gram-positive bacteria [24], [25], fungi [26], [27], and viruses [28], [29], [30]. Silver ions can inhibit microbial biofilm formation [27] and destabilize the polymeric matrix once the biofilm is formed [31]. This last finding could be of clinical relevance since vaginal infections are sustained by several microorganisms forming biofilm colonies [32], [33], [34].

In this *in vitro* study, we firstly assessed the antimicrobial activity of a novel organic sucrose octasulfate silver salt [C_12_H_14_Ag_8_O_35_S_8;_ IASOS, InterPharm Investments Limited, UK) against vaginal pathogens isolated from clinical cases of human symptomatic vaginitis. To this end, several *in vitro* assays were performed using different growth culture media and incubation pH values. Afterward we tested the antimicrobial properties of an innovative vaginal gel (SilSOS Femme) containing IASOS among the active ingredients. Other selected active ingredients in the formulation of the whole vaginal gel included KSOS (potassium sucrose octasulfate) for its trophic and anti-bacterial adhesion properties [35], [36], and sodium hyaluronate (HYA) for its strong moisturizing and healing promotion activities [37], [38].

## Materials and Methods

### Vaginal Pathogens and Growth Conditions

Six vaginal pathogens isolated from human clinical cases of symptomatic vaginal infections, namely: *Escherichia coli*, *Enterobacter aerogenes, Staphylococcus aureus*, *Streptococcus agalactiae*, *Gardnerella vaginalis* and the yeast *Candida albicans*, were included in our study. All strains were cultivated on Brain Heart Infusion (BHI) (Oxoid, England) agar plates; for *G. vaginalis* the medium was supplemented with 10% fetal bovine serum. Single colonies were picked up, transferred to BHI broth, and incubated at 37°C in aerobic atmosphere for 24 h. All grown colonies were characterized biochemically and molecularly by 16S rDNA sequencing, and stored in fresh BHI broth with 30% (vol/vol) glycerol at −20°C for subsequent experiments.

### Antimicrobial Activity of the IASOS

IASOS antimicrobial activity was tested by the Microtiter plate method, using dose-decreasing aqueous preparations of a starting solution containing 20 g/L silver ions, diluted either in BHI (1.75% brain heart extract, 1% peptone, 2% glucose, 0.5% NaCl and 0.25% phosphate buffer, supplemented with 10% fetal bovine serum for *G. vaginalis* only), or LAPT, a salt free medium (1.5% peptone, 1% triptone, 1% glucose, 1% yeast extract, 0.1% Tween-80). Each liquid medium was adjusted to pH values of 4, 4.5, 5 and 5.5 by the addition of HCl or NaOH as needed, and then autoclaved. In each of the media, the vaginal pathogens to be tested were sub-cultured three times at 37°C for 24 h to allow them to adapt to new environmental conditions. Fresh BHI or LAPT medium, at different starting pH values (4, 4.5, 5, and 5.5) were placed in 96-well plates, inoculated with 10^5^ CFU/ml of each pathogen and co-incubated with IASOS to achieve a final silver concentration of 10, 25, 50, 150 or 200 mg/L into the well. Optical Density (OD) measurements at 630 nm were carried out at baseline (OD_0_) and at 24 h (OD_24_) of incubation, and the kinetic analysis of microorganism growth (ΔOD) estimated. IASOS antimicrobial activity was expressed as percent reduction in pathogen growth, as compared to silver-free control cultures. Each experiment was performed in triplicate. All procedures were performed inside a dark cabinet to prevent any chemical changes from occurring when ionic silver is exposed to light. Two independent experiments were performed in triplicates.

### IASOS Microbiocidal and Microbiostatic Assays

Once a ΔOD was observed in the Microtiter plate method, the standard plate count was the applied quantitative assays to differentiate between a IASOS- microbiocidal or microbiostatic activity. One volume of each 24-h cultured pathogen was mixed into a tube with one volume of fresh BHI broth at pH 5 supplemented with IASOS, to achieve a final silver concentration of 50, 100 or 200 mg/L. Controls were prepared mixing each 24-h cultured pathogen with unsupplemented broth. Tubes were incubated in the dark at 37°C for 24 h. The mixtures were then centrifuged at 15,000×g for 5 min. The pellets were washed once and re-suspended in saline solution. Suspended cells were serially diluted and plated by pouring on the surface of BHI agar plates. After 24-h incubation at 37°C, colonies were counted. A 3 log reduction from the initial count was taken as a bactericidal activity.

### SilSOS Femme Vaginal Gel

SilSOS Femme (SF) (CM&D Pharma Limited, UK) is an aqueous gel containing IASOS 100 mg/L (i.e. 100 ppm). Other active ingredients in the formulation are KSOS and HYA, whereas propylene glycol and Carbopol Ultrez 10 (Lubrizol) are excipients and gel-forming thickener, respectively. To assess the differential contribution of IASOS (*versus* KSOS and HYA) to the antimicrobial properties of the whole vaginal gel, a formulation containing all the active ingredients but IASOS (I-SF) and a formulation containing excipients but no active ingredients (PL) were prepared for comparative analysis.

### Antimicrobial Activity of the Gels

Due to gel viscosity, the antimicrobial activity of the gel formulations had to be tested on solid agar plates. In a first set of experiments, different amounts of SF, I-SF, PL were spread onto the entire surface of 90 mm diameter, 10-ml BHI agar plates, to achieve the final concentration of 1, 2, 4, 8, 16, 32 mg/cm^2^. In parallel, plates containing the corresponding amount of Ag^+^ from IASOS were also run. All plates were left to dry and then poured with 5 ml BHI medium previously inoculated with 10^5^–10^6^ CFU ml^–1^ of the pathogen *E. coli* or *C. albicans*. Plates were allowed to solidify, incubated in the dark at 37°C for 24 h under anaerobic conditions and then macroscopically evaluated for opacity of surface growth, described as opaque, transparent or translucent (partially transparent) depending on the degree of growth. Plates containing no gel formulation and those containing BHI agar only, were used as controls.

In a second set of experiments, 32 mg of SF, I-SF and PL were spotted in an area of 1 cm^2^directly on the surface of BHI agar plates previously adjusted to pH 5 and spread with 100 µl of a 24-h culture of each pathogen. The equivalent amount of silver ions contained in 32 mg of SF, that is 3.2 µg of silver ions, was tested by dropping directly the IASOS over 1-cm^2^ area of the agar surface. In addition, 32 mg of I-SF or PL were supplemented with 3.2 µg each of ionic silver by using the proper dilution of IASOS (I-SF+Ag^+^, and PL+Ag^+^, respectively), and tested as above. Each experiment was carried out in duplicate and repeated twice. All plate were incubated in the dark at 37°C for 24 h and then observed. After incubation, plates were evaluated for any sign of growth inhibition (halo) around the pathogen spots.

### Statistical Analysis

Results of the antimicrobial activity of IASOS in liquid media are expressed as the mean of two independent experiments performed in triplicates ± standard deviation.

## Results

### Antimicrobial Activity of the IASOS

**Figure 1:**
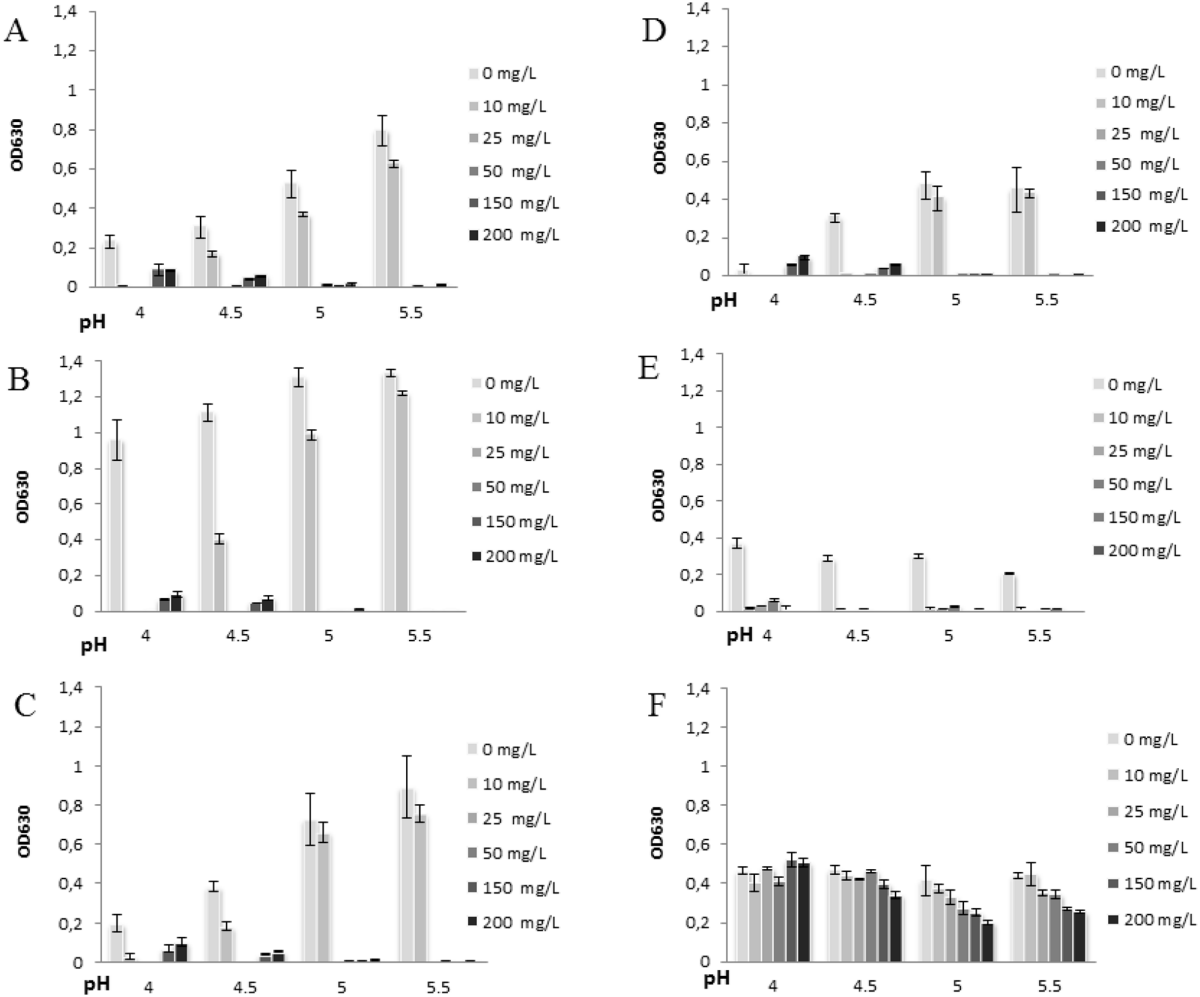
Growth curves obtained in BHI broth at different final silver concentrations and at different starting pH values. Each bar represents the mean of 24(ΔOD), across two independent experiments performed in triplicates ± standard deviation: A) *Escherichia coli*, B) *Enterobacter aerogenes*, C) *Staphylococcus aureus*, D) *Streptococcus agalactiae*, E) *Gardnerella vaginalis*, and F) *Candida albicans.*

**Figure 2:**
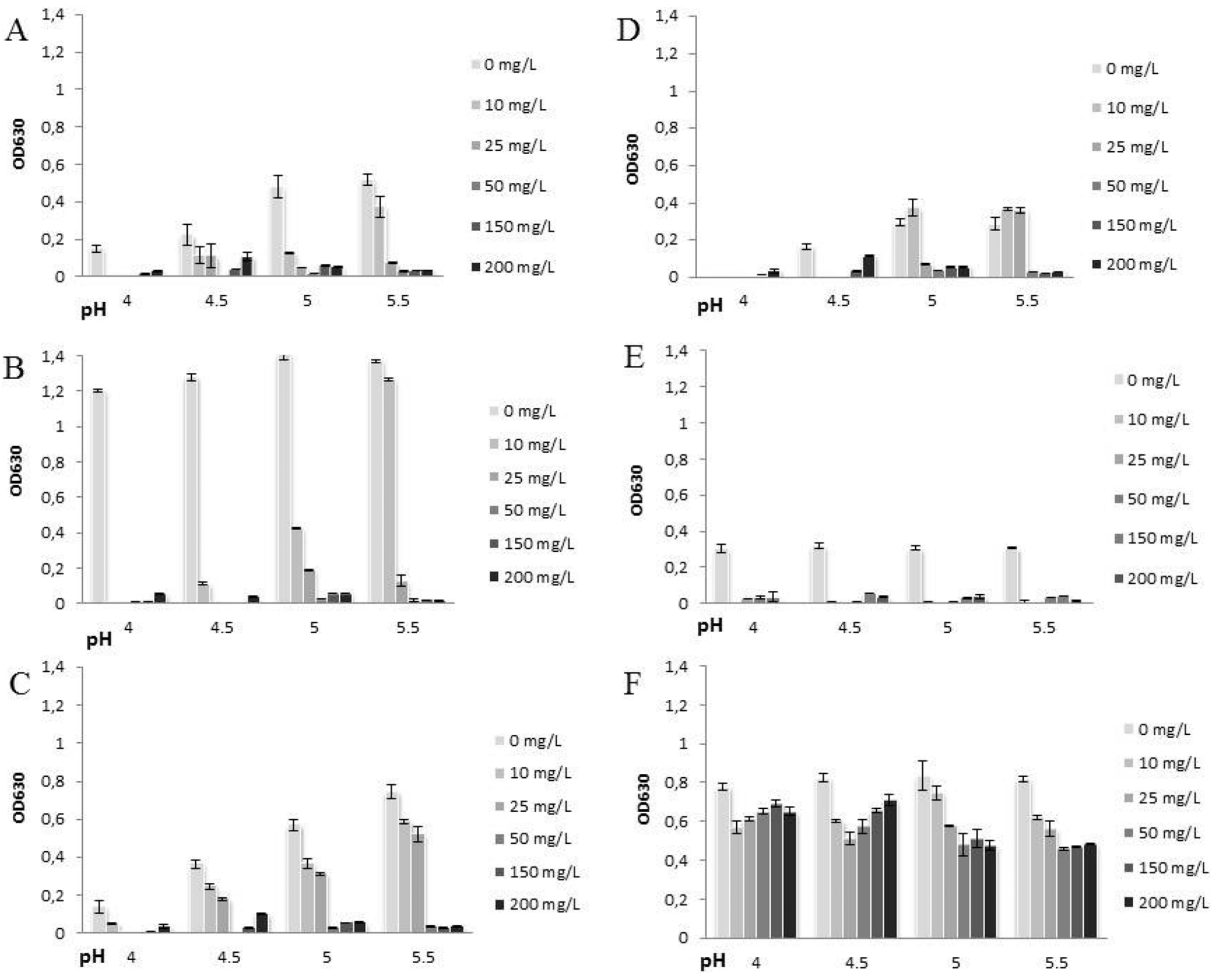
Growth curves obtained in LAPT broth at different final silver concentration and at different starting pH values. Each bar represents the mean of 24(ΔOD), across two independent experiments performed in triplicates ± standard deviation: A) *Escherichia coli*, B) *Enterobacter aerogenes*, C) *Staphylococcus aureus*, D) *Streptococcus agalactiae*, E) *Gardnerella vaginalis*, and F) *Candida albicans.*

In control plates, BHI and LAPT broths supported microbial growth of all the tested pathogens with the highest efficiency at pH 5–5.5, generally. When IASOS was added to the cultures, a strong antimicrobial activity was observed. Figures 1 and 2 show the growth inhibitory effect of IASOS (expressed as ΔOD) in cultures prepared with different media, different starting pH values and different final silver ion concentrations.

In both BHI and LAPT broth, IASOS resulted effective in inhibiting bacterial growth with an almost complete growth arrest against all the pathogens tested at 50–200 ppm Ag**^+^**. The effect was particularly evident at pH 5–5.5. At the lowest silver ion concentrations (10–25 ppm Ag^+^), microbial strains were also differentially inhibited in growth, depending on the broth and the pH used. In BHI, the Minimal Bactericidal Concentration of silver ions (MBC), i.e. the minimal concentration totally inhibiting growth, were 10 mg/L for *G. vaginalis* and 25 mg/L for the other bacterial pathogens. In LAPT medium, the MBCs were 10 mg/L for *G. vaginalis*, 25 mg/L for *E. coli* and *E. aerogenes*, and 50 mg/L for *S. aureus* and *S. agalactiae*, respectively.

Under all the experimental conditions tested, total growth inhibition was never observed for *C. albicans.* In BHI broth and at pH 5–5.5, *C. albicans* was significantly inhibited by IASOS concentration corresponding to 50, 150, 200 ppm Ag^+^. 200 ppm Ag^+^ induced the highest degree of inhibition (ΔOD: −52%) at pH 5. In LAPT medium and at pH 5–5.5, *C. albicans* was inhibited (ΔOD: −40%) by 50, 150 or 200 mg/L Ag^+^. A milder but still significant inhibition (ΔOD: −34%) was observed at 25 mg/L Ag^+^, at pH 4.5. Worth noting that at the final concentration of 10 mg/L Ag+, a significant antimicrobial activity of IASOS against *C. albicans* (yeast growth reduction: −27%) was observed in LAPT at pH 4 and 4.5. In BHI this dose was completely ineffective.

Due to the very small differences observed in microbial growth between BHI and LAPT media, BHI medium only was used in all subsequent experiments. Besides, a starting pH value of 5 was chosen to mimic the vaginal environment typical of many bacterial infections.

### Microbiocidal and Microbiostatic Assays

The standard plate count assay differentiated the microbiocidal and microbiostatic activity of IASOS. A strong antimicrobial activity was observed against all bacterial pathogens at almost all Ag^+^ concentrations. Conversely, one logarithmic drop in cell number was observed for *C. albicans* (from 10^5^ CFU/ml in controls to 10^4^ CFU/ml in cultures with silver ions) at all tested final Ag^+^ concentrations. Therefore and under our experimental conditions, IASOS produced a biocidal activity against the tested bacteria and a microbiostatic effect against *C. albicans*.

### Antimicrobial Activity of the Different Gel formulations

Table 1 summarises data on the antimicrobial activity of the commercial vaginal gel formulation (SF) when compared with I-SF (missing IASOS in the formulation), and with PL (excipients only in gel formulation). In this first set of experiments, increasing amounts of gels (mg/cm^2^) were spread on the surface of agar plates to detect *E. coli* and *C. albicans* growth inhibition by translucency. 32 mg/cm^2^ (i.e. 2080 mg per 90 mm Ø Petri dish) of SF was the lowest concentration capable to affect *E. coli* and *C. albicans* surface growth, and therefore defined as the cut-off concentration in further experiments.

**Table 1:** Effects of the different vaginal gel formulations and IASOS on *E. coli* and *C. albicans* growth.

Vaginal gel amount spread over plates mg/cm^2^ (total/plate)	Pathogen surface growth* in presence of the gel			Amount of IASOS spread over the plates (expressed as Ag^+^)	Pathogen surface growth* in presence of IASOS
	SF	I-SF	PL		
1 mg/cm^2^ (65 mg)	−	−	−	6.5 µg	−
2 mg/cm^2^ (130 mg)	−	−	−	13 µg	+/−
4 mg/cm^2^ (260 mg)	−	−	−	26 µg	+/−
8 mg/cm^2^ (520 mg)	−	−	−	52 µg	+/−
16 mg/cm^2^ (1040 mg)	−	−	−	104 µg	+/−
32 mg/cm^2^ (2080 mg)	+	−	−	208 µg	++

*pathogen surface growth: completely opaque and equal to controls (−), slightly reduced in opacity (+/−), translucent (+), almost totally transparent (++); SF = SilSOS Femme; I-SF = gel formulation containing all the active ingredients but IASOS; PL = gel formulation containing excipients but no active ingredients.

No antimicrobial activity was instead detected for I-SF and PL at any mg/cm^2^, confirming that the antimicrobial activity of the gel was strictly dependent on the presence of IASOS in its formulation. As a proof of concept, in the plates run in parallel and spread with the corresponding amounts of IASOS (expressed as silver ions, Ag^+^), an inhibitory effect on microbial growth was observed at all IASOS concentrations tested, with the only exception of 6.5 µg Ag^+^. At 200 µg Ag^+^, IASOS almost totally inhibited the pathogen surface growth.

In a second set of experiments SF, I-SF and PL antimicrobial activity was assessed against all the vaginal pathogens tested by spotting the 32 mg/cm^2^ cut-off concentration on 1 cm^2^ plate surface. Again, plates spotted with IASOS were run in parallel, at an equivalent amount of silver ions (Ag^+^) as per those contained in 32 mg of gel. Zones of growth inhibition were observed for all pathogens when IASOS or SF were spotted over the agar surface, although differences induced by IASOS and SF in the boundaries and size of inhibition haloes were observed among the various pathogens. Clear inhibition zones with regular boundaries and of similar size were observed for *S. agalactiae* and *G. vaginalis*; clear areas of inhibition with irregular boundaries were observed for *E. aerogenes*; few colonies growing within IASOS but not within SF zone were observed for *S. aureus*. Neither SF nor IASOS completely inhibited the growth of *E. coli* and *C. albicans* after 24 h incubation. I-SF and PL did not inhibit the growth of the pathogens. Interestingly, when both I-SF and PL were supplemented with the proper amount of IASOS (I-SF+Ag^+^ and PL−Ag^+^, the antimicrobial effect was restored and no differences were observed between the haloes formed by SF, I-SF+Ag^+^ and PL−Ag^+^ for each pathogens (data not shown).

## Discussion

The present study assessed *in vitro* the antimicrobial activity of a novel silver organic salt, IASOS, and of the innovative vaginal gel SilSOS Femme, containing IASOS as active ingredient against pathogens isolated from vaginal swabs of women symptomatic for vaginitis. Both IASOS and SilSOS Femme inhibited the growth of all the tested pathogens in the two culture media used, BHI and LAPT. The extent of IASOS antimicrobial efficacy seemed to depend on pathogen strain, silver ions concentration, and pH.

To verify the nature of IASOS antimicrobial behaviour, its microbiocidal and/or microbiostatic property was studied in three different concentrations of silver ions (50, 100 and 200 mg/L). In our experimental conditions, all the three concentrations displayed a bactericidal action against all the bacteria tested, whereas a fungistatic effect could be observed against *C. albicans*. These results are in agreement with the Microtiter plate assay performed in BHI, where a complete inhibition of bacterial growth was found at final silver ionic concentrations ≥25 mg/L, and a 35–52% decrease in yeast growth at final silver ions concentration ≥50 mg/L. Our findings suggest that the bactericidal and fungistatic effect of IASOS is dependent on both ionic silver concentration and microorganism, in terms of species and initial inoculum.

The pH is a critical environmental parameter for the regulation of the vaginal ecosystem. An imbalance of the ordinary vaginal microbiota usually causes vaginal pH to increase over 4.5, thereby creating a favorable local milieu to the growth of potentially pathogenic bacteria [39]. To the best of our knowledge, no studies investigating the effect of the pH on the silver antimicrobial efficiency are available in literature. We found that pH may indeed affect the antimicrobial activity of IASOS (Figure 1 and 2). IASOS generally showed a stronger antimicrobial activity at pHs higher than the physiological pH 3.8–4.5, at final ionic silver concentrations higher than 10 mg/L. More evident in BHI medium for all the pathogens tested, this observation is clinically relevant since a vaginal pH>4.5 is an usual finding in patients affected by bacterial vaginosis and aerobic vaginitis, being also a diagnostic parameter [5], [40]. The overall results suggest that the effects of pH on IASOS efficacy depend on the final concentration of available silver ions, the conditions of microbial growth, and the pathogenic species involved.

Differently from theoretical expectations, the influence of salts and organic matter in the culture media was of minor relevance. Organic matter and salts present in the culture media could interfere on the antimicrobial activity of silver ions because of their propensity to form complexes with silver ions, thereby reducing silver ions availability to exert the desired antimicrobial activity [41]. Instead, only slight differences were found in the antimicrobial activity of IASOS in relation to the two different media used. These differences were limited to a lower antimicrobial activity against *S. aureus* and *S. agalactiae* in LAPT, resulting in both cases in a higher MBC (50 mg/L silver ions in LAPT *versus* 25 mg/L in BHI), and a stronger antimicrobial activity against *C. albicans* in LAPT. Comparable MBCs were instead found in both media for *G. vaginalis* (10 mg/L ionic silver) and for *E. coli* and *E*. *aerogenes* (25 mg/L ionic silver). The different composition in salts and organic compounds of the two media, as well as a diverse susceptibility to silver ions of the different pathogenic species, might explain these results. In support to the present observations, a previous study [41] also found an equal MBC for *E. coli* (25 mg/L ionic silver) and a higher MBC for *S. aureus* (200 mg/L ionic silver). Even if in that study chemically-produced silver nanoparticles were used as a source of silver ions and the determination of the MBCs were performed in different experimental conditions, the similarities of the results is relevant. In an another study, a surfactant-based, temperature-sensitive gel containing silver nanoparticles was tested; a MBC of 5 mg/L was found for *S. aureus* and MBCs of 10 mg/L were found for both *E. coli* and *Pseudomonas aeruginosa* [42]. In this study *G. vaginalis* resulted the most sensitive to IASOS activity in both media and for all pH tested (MBC  = 10 mg/L ionic silver). As far as we know no studies testing the susceptibility of *G. vaginalis* to silver ions are available in literature.

In our experimental conditions we could not find the minimal concentration totally inhibiting growth of *C. albicans*; nevertheless IASOS, in BHI cultures and at pH 5 and 5.5, was able to decrease yeast growth rate of 35%, 40% and over 42% (range between 42–52%) respect to controls, at concentration of 50, 150 and 200 mg/L silver ions, respectively. Reduced *C. albicans* growth was also observed in LAPT at pH 5 and 5.5; a decrease of about 34% was found at a concentration of 25 mg/L silver ions, while at higher concentrations (50, 150 and 200 mg/L silver ions) the reduction in yeast growth was as high as 40%.

According to previous experimental studies, the toxic effects of silver on *Candida* species depends on loss of membrane integrity and membrane potential, with consequent reduction of cell viability [43]. Furthermore, it has been recently shown that silver can also exert its antifungal effects against *Candida* biofilms. Colloidal silver nanoparticles has been reported to affect matrix composition and structure of Candida biofilms in a dose-independent manner [44], [45]. Silver is believed to exert antimicrobial properties against fungi, viruses and protozoa according to a mechanism of action similar to its antibacterial effects. Through binding to biological molecules containing thiol, amino, carboxylate, imidazole and phosphate groups, ionic silver forms inhibit vital activities of microbial enzymes and proteins such as ATP production [46] and glucose oxidation [47]. Silver ions can make complexes with microbial DNA and RNA [24], [48], and microbial membranes are damaged by silver-generated free radicals [49], [50], [51]. All these data indicate that silver, in proper conditions, can be an effective antifungal agent.

So as the antimicrobial activity of the vaginal gel SilSOS Femme is concerned, an amount as high as 32 mg per cm^2^ was needed to achieve a visible inhibition of microbial growth in our experimental conditions, suggesting that the vaginal gel could be less effective than the corresponding amount of Ag^+^ from IASOS. This discrepancy has to be ascribed to the viscous nature of the gel formulation, slowing down the diffusion of silver ions from the matrix, thereby apparently decreasing the amount of silver available for microbial growth inhibition. To this end, it is worth noting that the silver-free gel formulations resulted inactive against all pathogens. This finding suggests that all the other SF ingredients but IASOS do not exert antimicrobial activity. Moreover, when 32 mg of each gel formulation were spotted on 1 cm^2^ agar plates, SilSOS Femme and the corresponding amount of IASOS expressed as silver ions, were similarly able to form haloes against all the pathogens tested, whereas the silver-free gel formulations were totally unable to form inhibition haloes. The addition of the proper amount of silver ions to the silver-free gel formulations promptly recovered the antimicrobial activity, providing still evidence to the fact that IASOS-derived silver ions are responsible for the antimicrobial effects of SilSOS Femme. Nevertheless, SilSOS Femme displayed a stronger antimicrobial activity against *S. aureus*, *G. vaginalis* and *C. albicans*, well known to organize into biofilm colonies, making them resistant to several drug therapies [52]. This unexpected result could merit further assessment. The finding that different strains displayed differential sensitivity to IASOS alone or to SilSOS femme could suggest that other ingredients into the gel formulation, i.e. KSOS and HYA may synergize IASOS’s antimicrobial activity through their antiadhesion properties [36].

Although we were not able to assess, in our experimental conditions, the complex and dynamic host-bacteria relationships, which include biofilm formation, host factors, pathogen-resident microbiota interactions, etc., we were however able to evaluate *in vitro* the potential effectiveness of the antimicrobial agent IASOS against vaginal pathogens.

In conclusion, our *in vitro* results highlight the antimicrobial activity of IASOS, the patented silver salt of sucrose octasulfate, against a number of bacterial strains involved in the onset of symptomatic vaginitis, as well as against the yeast *C. albicans*, responsible of one of the most frequent vaginal fungal infection. The data obtained in this study indicate also that SilSOS Femme, the vaginal gel formulation containing IASOS, may display important antimicrobial effects against bacterial and yeast pathogens, suggesting a potential role of this novel formulation in the prevention and/or treatment of feminine vaginal infections as an adjunct to drug therapy.
